# Understanding the Adult Mammalian Heart at Single-Cell RNA-Seq Resolution

**DOI:** 10.3389/fcell.2021.645276

**Published:** 2021-05-12

**Authors:** Ernesto Marín-Sedeño, Xabier Martínez de Morentin, Jose M. Pérez-Pomares, David Gómez-Cabrero, Adrián Ruiz-Villalba

**Affiliations:** ^1^Department of Animal Biology, Faculty of Sciences, Instituto Malagueño de Biomedicina, University of Málaga, Málaga, Spain; ^2^BIONAND, Centro Andaluz de Nanomedicina y Biotecnología, Junta de Andalucía, Universidad de Málaga, Málaga, Spain; ^3^Traslational Bioinformatics Unit, Navarrabiomed, Complejo Hospitalario de Navarra, Instituto de Investigación Sanitaria de Navarra (IdiSNA), Universidad Pública de Navarra, Pamplona, Spain; ^4^Centre of Host-Microbiome Interactions, Faculty of Dentistry, Oral & Craniofacial Sciences, King’s College London, London, United Kingdom; ^5^Biological and Environmental Sciences and Engineering Division, King Abdullah University of Science and Technology, Thuwal, Saudi Arabia

**Keywords:** single-cell RNAseq, heart, infarction, cardiac cell heterogeneity, transcriptomics

## Abstract

During the last decade, extensive efforts have been made to comprehend cardiac cell genetic and functional diversity. Such knowledge allows for the definition of the cardiac cellular interactome as a reasonable strategy to increase our understanding of the normal and pathologic heart. Previous experimental approaches including cell lineage tracing, flow cytometry, and bulk RNA-Seq have often tackled the analysis of cardiac cell diversity as based on the assumption that cell types can be identified by the expression of a single gene. More recently, however, the emergence of single-cell RNA-Seq technology has led us to explore the diversity of individual cells, enabling the cardiovascular research community to redefine cardiac cell subpopulations and identify relevant ones, and even novel cell types, through their cell-specific transcriptomic signatures in an unbiased manner. These findings are changing our understanding of cell composition and in consequence the identification of potential therapeutic targets for different cardiac diseases. In this review, we provide an overview of the continuously changing cardiac cellular landscape, traveling from the pre-single-cell RNA-Seq times to the single cell-RNA-Seq revolution, and discuss the utilities and limitations of this technology.

## Introduction

The heart is the first fully functional organ forming during embryonic development [Hamburger and Hamilton stages (HH) 9–10 in the chicken, at embryonic day (E) 8 in the mouse, and Carnegie stages (CS) 9–10 in the human] (Hamburger and Hamilton, 1951; Theiler, 1972; Müller and O’Rahilly, 1987). In mammals, the cardiac primordium starts beating very soon (around E8.5 in the mouse, and between 26 and 32 days after conception in the human) (Howe et al., 1991; Porter and Rivkees, 2001), and progressively transforms into a four-chambered heart that does not stop its contractile activity until the death of the organism (Buckingham et al., 2005). Coordination is, no doubt, one of the most remarkable features of cardiac cell function. Indeed, cardiac pumping activity requires the continuous interaction of a great variety of cell types, including contractile cardiomyocytes, endocardial cells, vascular endothelial and smooth muscle cells, cardiac fibroblasts and pacemaker cells, among many others. Cardiac cell interactions, mediated by juxtacrine, paracrine and endocrine signals, sustain cardiac homeostasis and are essential to articulate heart responses to pathologic stimuli (Buckingham et al., 2005; Rog-Zielinska et al., 2016; Pogontke et al., 2019). These interactions are, at least in part, modulated by the specific functional profile of different cardiac chambers (e.g., intra-chamber pressure and resistance to blood flow are significantly different in the left versus the right ventricle). Moreover, in a pathologic context, cardiac function can be severely modified. Such functional changes may result from altered inter-cellular signaling, loss of cell activity or cell death, but it is also evident that changes in cardiac performance can then result in further changes in cell-to-cell communication.

Surprisingly, our knowledge of cardiac cell diversity in the mammalian heart has remained limited, which hampers our understanding of the heart as a complex cellular system requiring the finely orchestrated and accurate activity of its cellular components. Quantifying cardiac cellular components was always a goal of classical organographists (Roberts and Wearn, 1941), while researchers studying cardiac embryonic development have provided a continuous stream of relevant information about the cell types that build up the heart. Cardiogenesis, which involves the patterned differentiation of different cell types from multiple embryonic heart fields (Buckingham et al., 2005; Srivastava, 2006; Dunwoodie, 2007; Meilhac and Buckingham, 2018), is a unique context for the study of cardiac cell diversity. The genuine interest of cardiac embryologists in understanding the diversification of cardiac cell types from their mesodermal progenitors has generated a considerable volume of research in this field over the last two decades.

Cardiac cell differentiation and diversification have been studied using a great variety of cell tracing techniques in different experimental models. Genetic cell tracing methods, based on the known specific activity of one or several genes in certain cell types, have been extensively used. Unfortunately, this approach often involves the use of one gene at the time. Therefore, the complexity underlying a specific cell lineage is unlikely to be revealed using such methods, as we will further discuss in this review (see Meilhac and Buckingham, 2018).

Gene expression profiling at single-cell RNA sequencing (scRNA-Seq) resolution is a revolutionary and robust high throughput technology that allows for the understanding of complex biological systems from the analysis of their components via the unbiased identification of new cell types, cell lineage progression, and cellular plasticity in dynamic processes (Massaia et al., 2018; Meilhac and Buckingham, 2018; Mereu et al., 2020). One of the most relevant properties of this technology is its capacity to identify cellular heterogeneity at single cell resolution as based on the prevalence and/or co-expression of genes. On the other hand, scRNA-Seq allows for the unbiassed approach to the massive analysis of gene expression, and has a remarkable ability to compare data from several species in an integrative manner. Moreover, due to its excellent resolution power, scRNA-Seq is breaking new ground in several biological disciplines (Stubbington et al., 2017; Zheng et al., 2017; Xia and Yanai, 2019; Imdahl and Saliba, 2020; Jakab and Augustin, 2020; Rich-Griffin et al., 2020; Zhou and Wang, 2020). In fact, the use of scRNA-Seq technology constitutes a milestone in the history of research on the human body, leading to the generation of a human organ atlas at single-cell resolution (Haber et al., 2017; Han et al., 2018; Vanlandewijck et al., 2018; Aizarani et al., 2019; Kalucka et al., 2020; Litviňuková et al., 2020; Travaglini et al., 2020).

In the context of cardiac research, the high resolution of scRNA-Seq technology has been key to the dissection of cardiac cell diversity. Such diversity is evident from the early stages of cardiac development. Indeed, the precardiac mesoderm has been shown to be heterogeneous, comprising cells characterized by the activation of significantly different transcriptional programs that affect their developmental fate. Early in mammalian development, cardiac precursors organize into two cardiac progenitor domains (the First Heart Field, FHF and the Second Heart Field, SHF) that sequentially contribute to the formation of the embryonic cardiac primordium. FHF cells characteristically express the transcription factors Nkx2.5, Mef2C, Srf and Gata4, whereas SHF cells can be primarily identified by the expression of the transcription factors Isl1 and Tbx1 (Kelly et al., 2014). Multiple studies have characterized the properties of these two morphogenetic fields, it has been only since the advent of scRNA-Seq approaches that we have really started to understand the real heterogeneity of these cell populations. Lescroart et al. (2018) have recently identified that the first cardiovascular progenitor cells (CPCs), Mesp1^*POS*^ cells derived from the primitive streak around E6.5, are heterogeneous at scRNA-Seq resolution. This study provides robust data indicating that the capacity of these cells to differentiate into specific cardiovascular lineages is temporal and spatial determined at early gastrulation stages (Lescroart et al., 2018). In the same line, Jia et al. (2018) recently described three Nkx2.5^*POS*^ and five Isl1^*POS*^ different subpopulations of CPCs with significant differences in their trajectorial patterns and chromatin accessibility, two features that are thought to determine their differentiation capacity. These two studies highlight the enormous complexity that underlies the regulation of decisions during heart development, and are a good example of the remarkable resolution of scRNA-Seq analysis even when dealing with small number of cells.

In this review, we summarize current knowledge regarding the heterogeneity of the main cellular components of the adult mammal heart and their dynamics in both healthy and pathological conditions. In order to do so, we compare the vision of cardiac cell composition before and after the appearance of scRNA-Seq technology. In addition, we also describe the main drawbacks and limitations of this powerful tool and discuss the future perspectives it opens in the cardiovascular research field.

## Cellular Landscape of the Adult Heart Before Single Cell RNA-Seq Technology

The systematic analysis of cardiac cell composition was initiated during the first half of the 20th century (Roberts and Wearn, 1941). Using stereological and morphometric approaches in adult rats, cardiomyocytes (CMs) were found to cover 75% of the total volume of the heart, although it soon became clear they accounted for 25-43% of the cardiac cells only (Zak, 1973; Anversa et al., 1980; Nag, 1980). Some years later, an important study based on flow cytometry and immunohistochemistry suggested that significant difference in the cellular composition of the mouse versus the rat heart existed (26.4 ± 5.8 vs. 55.9 ± 8.3% of CMs, respectively) (Banerjee et al., 2007). Notwithstanding this, a consensus was reached on this topic, and cardiomyocytes were estimated to represent around 30% of total cardiac cells (Walsh et al., 2010; Bergmann et al., 2015). In contrast with studies on CMs, research on non-myocyte cells, including cell types as important as cardiac fibroblasts and endothelial cells, has remained scarce and inconclusive.

The careful evaluation of these studies suggests that their different conclusions can be accounted for by their extensive use of histological technologies to quantify cell numbers (Zak, 1973; Vliegen et al., 1991; Banerjee et al., 2007; Tang et al., 2009). To overcome the bias inherent in histological approaches and the occasional lack of specificity of markers for specific cell types, cell lineage tracing methods in combination with other technologies were used. Primarily, cell-tracing techniques, ranging from the use of carbon particles to auto-radiographic tattooing and advanced fluorescent dyes, were applied to describe cell migration or conformational changes in the developing heart (Castro-Quezada et al., 1972; Thompson et al., 1987; Patwardhan et al., 2000). Unfortunately, these methods frequently lack single-cell resolution, and provide cell tagging that can be lost over time (e.g., by dilution of the dye). Replication-defective retroviruses have also been effectively used to trace the origin and differentiation of cardiac cells (Mikawa and Fischman, 1992; Mikawa et al., 1992), but the retrovirus production process, and the need for highly accurate local viral delivery, limit the scope of this method. Paradoxically, lessons from the experimental use of retroviruses to label cells using viral genome integration in the host cell DNA strongly boosted the use of transgenic mouse lines to unambiguously track and identify cells.

The first transgenic mouse lines generated to tag cardiovascular cells were based on constructs containing a reporter cassette (most typically *LacZ* or *GFP*) under the control of a specific gene promoter sequence (Fadel et al., 1999; Didié et al., 2013; Velecela et al., 2013). In these animal models, the reporter expression is dependent on tissue/cell type-specific promoter activity, thus preventing the permanent genetic tracing of cell progenies. The advent of the Cre/LoxP (Soriano, 1999) or Tet OFF (Harding et al., 1998) genetic technologies addressed this issue, and the number of studies using these methods to study cell progenies increased enormously. Genetic cell tracing methods are extremely powerful and still have a deep impact on our understanding of heart cell composition, structure, and responses to pathologic stimuli (Moses et al., 2001; Huynh et al., 2007; Engleka et al., 2012; Ruiz-Villalba et al., 2015; Cano et al., 2016). However, these technologies have drawbacks, such as the association of a specific cell type with the expression of a single gene or the erroneous interpretation of the concept of cell lineage as based on the expression of such a gene (for an authoritative review on this topic see Meilhac and Buckingham, 2018).

Most cardiomyocyte subtypes are anatomically patterned. Contractile working CMs present in the left ventricle are mainly derived from cardiac progenitors of the first heart field, whereas the principal origin of right ventricular working CMs is the secondary heart field (Kelly et al., 2014; Meilhac and Buckingham, 2018). Endothelial cells (ECs) in the coronary vascular system mainly originate from the sinus venosus and ventricular endocardium (Red-Horse et al., 2010; Wu et al., 2012), with the contribution of septum transversum/epicardium (Cano et al., 2016) and other extracardiac endothelial cells (Palmquist-Gomes et al., 2018). The vast majority of cardiac ECs occupy the cardiac interstitium, a unique harboring compartment hosting a large variety of cells (Pogontke et al., 2019). Cardiac fibroblasts (CFs) are prototypical interstitial cells, and most CFs derive from the embryonic epicardium (Wessels et al., 2012; Moore-Morris et al., 2014; Ruiz-Villalba et al., 2015; Kanisicak et al., 2016), but endocardial-derived fibroblasts have also been described (Zeisberg et al., 2007). The cardiac interstitium is also the home of blood-borne cells that are recruited to the heart during embryonic development and postnatal stages. These cells, which will become cardiac resident, include different populations of monocytes/macrophages (Epelman et al., 2014; Ruiz-Villalba et al., 2015; Dick et al., 2019; Sampaio-Pinto et al., 2020).

In order to characterize and quantify cardiac cell composition, a sophisticated study was carried out using a FACS-based approach in murine and human hearts (Pinto et al., 2016). In this paper, the authors suggest that ECs are the most abundant cells in the adult heart, and that CFs and immune cells contribute less than 20% and 10%, respectively, to the non-myocyte cardiac cell fraction. These data differ from those discussed above, highlighting how differences in the experimental design of these type of analyses can result in significantly different results (Lescroart et al., 2012; Meilhac and Buckingham, 2018). It is anyway evident that all these studies have significantly helped to increase our knowledge of cardiac cell heterogeneity, especially in some experimental animal models like the mouse, even if this knowledge cannot be always extrapolated to humans.

In this regard, single-cell “-omics” appear as a set of revolutionary, unbiased methods allowing for the characterization of cardiac cell heterogeneity. In the last 5 years, the cardiovascular field has moved from the “cell-marker” concept, mostly used to define a “specific” cell type or lineage in the mouse, to the development of a first map draft of cardiac cell diversity based on single-nuclei RNA-Seq data and specifically annotated for the human heart (Litviňuková et al., 2020). In the following sections, we describe the contribution of scRNA-Seq analysis to (1) the state of knowledge about cardiac cell heterogeneity prior to the single-cell “-omics” era; and (2) how these techniques are significantly changing our understanding and experimental strategies to study the heart. To do that, we describe the most recent published scRNA-Seq data related to cardiac cell heterogeneity, both in mice and humans.

## Cardiomyocytes

**FIGURE 1:**
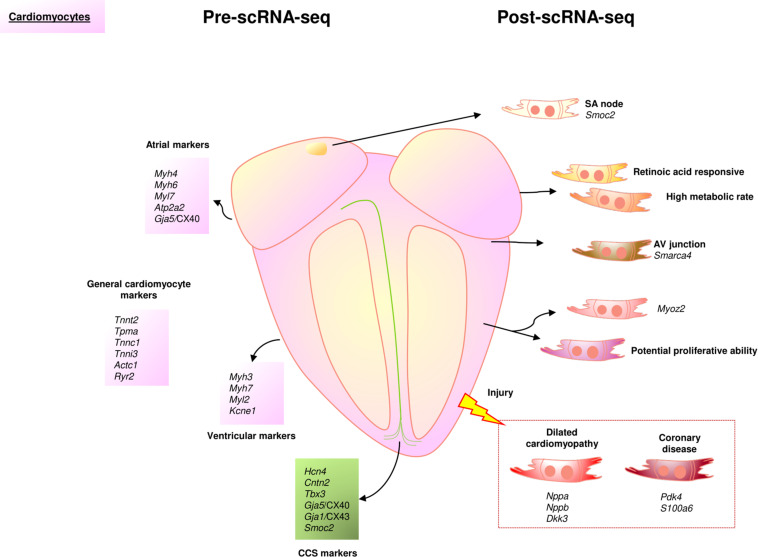
Cardiomyocytes landscape before and after scRNA-Seq. In the scRNA-Seq part, each cell represents an identified cluster characterized by one or more genes, a specific cell function or other cell aspects. *Nppa* and *Nppb* gene expression identifies characteristic cardiomyocyte subpopulations in the pathological context of dilated cardiomyopathy. CCS, cardiac conduction system; SA, sinoatrial; AV, atrioventricular.

Cardiomyocytes are required for cardiac contraction and the consequent distribution of blood throughout the organism. This crucial function is based on the coordinated depolarization of electrically coupled CMs in the atria and ventricles regulated by the cardiac conduction system (CCS) (Ng et al., 2010; van Eif et al., 2018). For this reason, CMs have been historically classified in terms of their contractile properties and anatomical location. In the pre scRNA-Seq era, CMs were identified as based on their expression of several common markers associated with the contractile machinery and coordination (*Tnnt2*, *Tpma*, *Tnnc1*, *Tnni3, Actc1, Ryr2*). However, atrial and ventricular compartments display significant differences in their gene expression profiles (Sehnert et al., 2002; Ng et al., 2010). Adult atrial CMs widely express the light and heavy myosin chains *Myh4*/ALC1 and *Myh6*/αMHC, whereas the light chain *Myl7*, the calcium regulator *Atp2a2*/SERCA2a, and the channel *Gja5*/CX40 present a more restricted expression. Ventricular CMs predominantly transcribe *Myh3*/ELC and *Myh7*/βMHC myosin genes, and specifically express *Myl2* and the potassium channel *Kcne1*. The transcriptional identity of each chamber has been associated with specific genes: *Hey1* in the atria and *Hey2* and *Irx4* in the ventricles (Ng et al., 2010; Figure 1).

The cardiac pacemaker or CCS is formed by specialized cells able to transduce the electrical stimulus that triggers cardiomyocyte contraction. These cells derive from primitive cardiomyocytes patterned in space to form a complex network of nodes (e.g., sinoatrial node; atrioventricular) and conduction tracts of different size (e.g., Bachmann’s bundle; bundle of His; right and left bundle branches; Purkinje fibers) (van Weerd and Christoffels, 2016). Primary functions of these different elements include the cyclic initiation of the electrical impulse (sinoatrial node), the fast propagation of the electric signal (CCS tracts or the delay of this same stimulus to guarantee atrial and ventricular systolic phases are asynchronous). Important parts of the CCS are protected by cardiac fibrous tissue, and recent studies have shown that macrophages play an important role in the normal functioning of the cardiac pacemaker (Anderson and Ho, 1998; Hulsmans et al., 2017). Adult CMs from the CCS have been traditionally identified as based on the expression of *Hcn4* (Liang et al., 2013) and *Cntn2*/Contactin-2 (Pallante et al., 2010). However, there are other markers such as *Gja1*/CX43, *Gja5*/CX40, *Smoc2*, *Isl1* (van Eif et al., 2019) or *Tbx3* (Hoogaars et al., 2004) that show a heterogeneous expression pattern within the different parts of the CCS (nodes, atrioventricular bundle, and the Purkinje fiber network) (Figure 1).

In this context, scRNA-Seq has significantly contributed to the study of cardiomyocyte heterogeneity. For example, different laboratories have recently identified a specific subpopulation of CMs characterized by enrichment in the expression of *Myoz2*, both in the adult murine and the developing human heart (Gladka et al., 2018; Asp et al., 2019). In adult human samples, transcriptomic profiling of CMs isolated from different anatomic compartments suggests functional specialization (Wang et al., 2020). Accordingly, small sets of CMs isolated from atria and ventricles showed significant differences in their metabolic activity, retinoic acid-responsive capacity and smooth muscle cell transcriptomic profiles (Litviňuková et al., 2020). Interestingly, the expression of *Smarca4*, a gene that defined the atrioventricular CM cluster, has been associated with cardiac hypertrophy (Wang et al., 2020). In contrast to their working myocardium counterparts, the single-cell identity of the adult CCS remains to be fully elucidated. Until now, scRNA-Seq has uncovered a complex landscape within cell-type heterogeneity and cellular transitional phenotypes in the human CCS during development (Goodyer et al., 2019). In the same study, the authors identified different cell populations in all the CCS components, and found that while the expression of *Igfbp5*, *Cpne5*, and *Ntm* is enriched in the entire CCS, *Smoc2* is exclusively expressed in the sinoatrial node.

Studies based on the scRNA-Seq technology have also contributed relevant data to the contentious topic of adult cardiomyocyte proliferation abilities. CMs have been convincingly shown to display some turnover during adult life (Bergmann et al., 2015). However, mammalian CMs are unable to regenerate the heart upon injury even if their proliferation under stress conditions seems to be significantly increased (Kimura et al., 2015). Proliferative CMs have been found in both embryonic and neonatal mouse hearts, but scRNA-Seq studies have been unable to identify proliferating cardiomyocytes, either in the adult homeostatic or the infarcted heart (Kretzschmar et al., 2018; Li G. et al., 2020). Only an integrative analysis using data from the “Tabula Muris” compendium (Schaum et al., 2018) and single-nuclei RNA-Seq (snRNA-seq) approaches has revealed the existence of a rare cardiomyocyte subpopulation (0,4% of total cardiac cells) displaying proliferative markers (Galow et al., 2020). Using the same single-cell technology, a previous report revealed that long intergenic non-coding RNAs were key regulators of the cell cycle in a subpopulation of CMs, suggesting the presence of CMs with an inherent proliferative ability in the adult heart (See et al., 2017). Taken together, these results emphasize that scRNA-Seq has not been able to definitively identify proliferative CMs. However, this technology can be used in combination with different high throughput technologies, such as ATAC-Seq, proteomics, or metabolomics, to properly address this search for future regenerative interventions (Hu et al., 2018; Kretzschmar et al., 2018; Li G. et al., 2020).

Deciphering the dynamics of cardiac cell populations upon injury has been another important aim of single-cell studies in the field. Hypertrophic CMs display high expression of the disease-related genes *Nppa*, *Nppb*, and *Vegfa* (Yekelchyk et al., 2019; McLellan et al., 2020). In the case of *Nppa* and *Nppb*, although their relevance in cardiac development and disease had been previously reported (Sergeeva et al., 2014), scRNA-Seq technology has revealed the transcriptomic signature that describes specific subpopulations of CMs involved in cardiac disease. Mono- and multi-nucleated myocytes have been shown to have similar transcriptional profiles in both homeostatic and pathological conditions (Yekelchyk et al., 2019). In human hearts with end-stage cardiac failure (dilated cardiomyopathy and coronary disease), a recent scRNA-Seq study has pointed out that the left ventricle is always the most severely affected compartment (Wang et al., 2020). Besides the commonly downregulated genes like *Spp1* or the transcription factors *Tcf7l2* and *Cebpd*, other differentially expressed genes (*Pdk4* and *S100a6* in coronary disease and *Nppa*, *Nppb*, and *Dkk3* in dilated cardiomyopathy) may help to identify specific mechanisms associated with these two cardiac diseases (Wang et al., 2020; Figure 1).

The analysis of CMs at single-cell resolution also has its drawbacks (Li G. et al., 2020). These include the use of protocols for tissue dissociation (which can damage and destroy cells) and the variable size of adult CMs, as this can alter cell capture in single-cell platforms (Gladka et al., 2018; Zhou and Wang, 2020). In any case, scRNA-Seq has been proven to be a very robust technology. Indeed, although the ploidy of CMs and the nuclear transcripts may affect the results obtained in these experiments (Zhou and Wang, 2020), similar transcriptional profiles were obtained when comparing data from single-cell and single-nuclei RNA-Seq (Selewa et al., 2020). Regarding heart composition, snRNA-Seq revealed that CMs represent between 23 and 49% of cardiac cells (Galow et al., 2020; Litviňuková et al., 2020; Tucker et al., 2020; Wolfien et al., 2020a, b) in contrast to the 9% indicated from the scRNA-Seq of the “Tabula Muris” project (Schaum et al., 2018). Different proportions of CMs have been found between the atria (30%) and ventricles (49%) in the human heart (Litviňuková et al., 2020), suggesting additional chamber-specific differences in cardiac cell distribution with functional implications. From a technical standpoint, we believe it is necessary to explore the limitations of scRNA-Seq in order to improve our understanding of cardiomyocyte diversity. This will require the simultaneous analysis of spatial gene expression patterns and the evaluation of the biological roles of genes, not only in homeostatic, but also in pathologic contexts.

## Endothelial Cells

**FIGURE 2:**
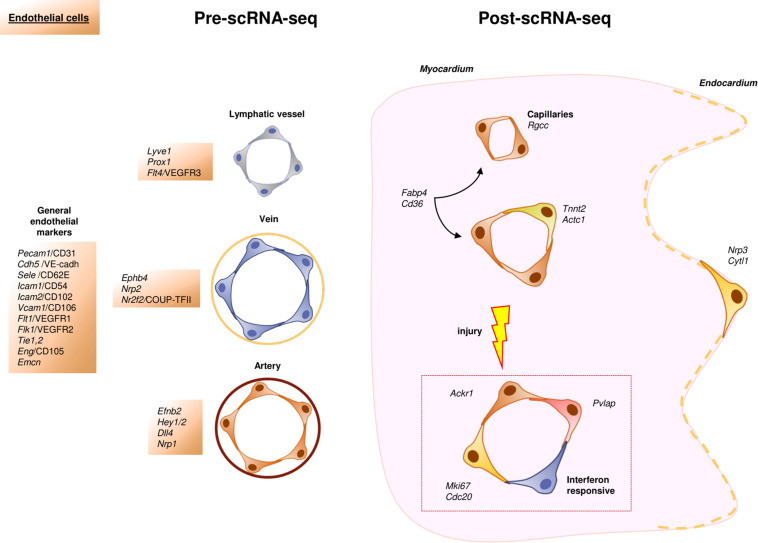
Schematic representation of the cardiac endothelial cellular landscape before and after scRNA-Seq. In the scRNA-Seq part, each color represents an identified cell subpopulation characterized by specific genes, cellular functions or other relevant aspects.

Cardiac ECs line the inner surface of cardiac chambers, blood and lymphatic vessels. They play a role in essential cardiovascular cell functions like permeability, leukocyte trafficking, hemostasis, thermoregulation or angiogenesis (Aird, 2007a; Talman and Kivelä, 2018). These cells display different morphological features across the vascular tree, but also respond to different gene expression programs (vascular versus lymphatic; arterial versus venous; and large versus microvascular endothelial phenotypes) (Chi et al., 2003; Nolan et al., 2013). Classically, the majority of endothelial markers found in the literature are related to the unique properties and functions of ECs, as is the case with *Pecam1*/CD31, *Cdh5*/VE-cadherin, *Sele*/E-selectin, *Icam2*/CD102, *Flt1*/VEGFR1, *Tie2*/Tek, *Eng*/CD105 (Garlanda and Dejana, 1997; Brutsaert, 2003; Aird, 2007a; Banerjee et al., 2012). Some other genes, however, have been found to be preferentially expressed in arterial (*Efnb2*, *Dll4*, *Hey1/2*, *Nrp1*), venous (*Ephb4*, *Nrp2*, *Nr2f2*/COUP-TFII) (Aird, 2007b), or lymphatic ECs (*Prox1*, *Lyve1, Flt4/*VEGFR3) (Wigle and Oliver, 1999; Mäkinen et al., 2001; Figure 2).

Despite the established knowledge on the heterogeneity of ECs, scRNA-Seq has allowed us to gain a deeper understanding of this complex population of cardiac cells. A very recent study has summarized the discoveries of endothelial cell diversity (or “angiodiversity” as the authors call it) resulting from the use of scRNA-Seq (Jakab and Augustin, 2020). Even though this technology has confirmed the role of some of the genes quoted above as markers for ECs, specific metabolic and gene expression programs were spotted and analyzed across the vascular tree. In a recent paper, Kalucka et al. (2020) have described several subsets of ECs with specialized phenotypes in the homeostatic heart, including capillary ECs with an interferon-induced gene program and some subpopulations with a characteristic angiogenic signature. Interestingly, the authors of this work also described how microvascular ECs were found to be the most heterogeneous endothelial cell pool within the same organ and even among different ones. Other authors proposed *Rgcc* as a specific marker for capillaries (Schaum et al., 2018; Kalucka et al., 2020). The endocardium, the special endothelium that covers internal cardiac chamber walls, is another interesting case, as it displays a high expression of *Npr3* and *Cytl1* (Feng et al., 2019), which are also expressed in some capillaries and arteries (Hu et al., 2018; Kalucka et al., 2020), and cannot be therefore regarded as endocardial markers. Interestingly, scRNA-Seq has confirmed previous bulk RNA-Seq data describing a specific transcriptomic signature for cardiac ECs (Coppiello et al., 2015). This signature includes genes involved in fatty acid uptake and metabolism like *Fabp4*, *Cd36*, *Pparg*, *Tcf15, Aqp7* and *Meox2* (Feng et al., 2019; Jambusaria et al., 2020; Kalucka et al., 2020), all of which could be relevant to our understanding of cardiomyocyte bioenergetics (Lother et al., 2018). In humans, some clusters of ECs were even identified on the basis of their propensity to secrete cytokines or their implication in immune response and cell-to-cell assembly (Wang et al., 2020; Figure 2).

Unlike CMs, ECs of adult hearts can proliferate (Kretzschmar et al., 2018). The use of scRNA-Seq technology has refined this concept by showing that a cluster of pre-existing ECs drives vasculogenesis in a clonal way in the healthy heart (Li et al., 2019). Rare subpopulations of ECs that co-express cardiomyocyte markers such as *Tnnt2* or *Actc1* have also been identified in data from sc/snRNA-Seq experiments (Hu et al., 2018; Nomura et al., 2018; Li et al., 2019; Lukowski et al., 2019; Galow et al., 2020; Jambusaria et al., 2020; Wolfien et al., 2020a). Whether these cells are involved in the modulation of the activity of CMs, as suggested by some authors (Jambusaria et al., 2020) is still unclear.

Single-cell RNA-Seq technology has also been used to study neovascularization phenomena after myocardial infarction (MI). Recent work has described the rise of several endothelial cell subsets enriched in proliferation markers (*Mki67*, *Cdc20*), showing either interferon or retinoic acid pathway signatures (Li et al., 2019). It is noteworthy that these “response to damage” endothelial subsets, enriched in *Plvap* expression, have been found to accumulate preferentially in the border zone of mouse and human infarcted hearts (Li et al., 2019). This finding led the authors to propose *Plvap* as a marker for neovasculogenesis and a target for future therapeutic approaches. Following a similar rationale, other groups have suggested that pre-existing pools of ECs are the primary source of new blood vessels in ischemic tissues (He et al., 2017; Manavski et al., 2018). As proof of concept, the administration *Ackr1*^+^ ECs, a subpopulation of ECs identified in healthy hearts exclusively, improved cardiac function when applied to a mouse infarct model. Because of that, these authors also considered these cells a potential therapeutic target to treat cardiac diseases (Wang et al., 2020).

## Fibroblasts and Mural Cells

**FIGURE 3:**
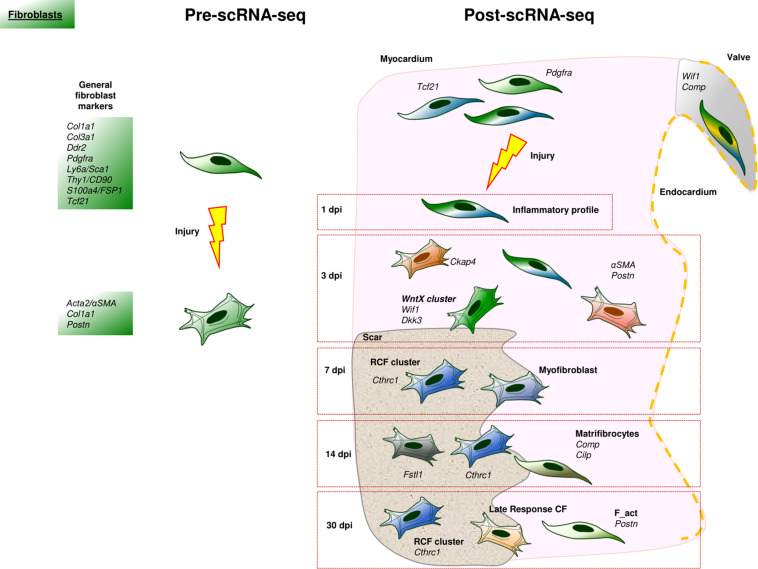
Cardiac fibroblast landscape before and after scRNA-Seq. In the scRNA-Seq part, each cell represents an identified subpopulation of cardiac fibroblasts characterized by a specific transcriptomic signature. Some of these populations have been named after the original study that identified them.

The study of CFs has been one major objective in the study of cardiac cells at single cell resolution. CFs are classically associated with the synthesis, deposition, and remodeling of the extracellular matrix (ECM). Moreover, they also contribute to cardiac homeostasis, communicate with immune cells, sustain cardiomyocyte electrical coupling and are essential to stress sensing (Camelliti et al., 2005; Souders et al., 2009; Frangogiannis, 2020). There is a set of “classical” markers defining CFs both under normal (CD90.1/THY1, PDGFRA, S100A4/FSP1, DDR2, SCA1, VIMENTIN, COL1A1) or pathological conditions (PERIOSTIN, αSMA*)*. However, most of these markers are not exclusive to CFs (Tallquist and Molkentin, 2017). For example, collagens are produced, to a certain extent, by many non-fibroblastic cells (Hynes, 2012; Fidler et al., 2017). Other molecules closely associated with CFs like VIMENTIN, CD90 or FSP1 are also found in endothelial and immune cells (Tallquist and Molkentin, 2017; Figure 3). As expected, the scRNA-Seq technology has enormously contributed to the identification and understanding of CFs heterogeneity in normal and pathological conditions.

First, scRNA-Seq has unequivocally confirmed that CFs are a heterogeneous cell type. Cardiac fibroblast populations can be characterized by their variable expression of classical markers such as *Pdgfra* and *Tcf21*, and different functions have been suggested for some of these fibroblast types (Kretzschmar et al., 2018; Skelly et al., 2018; Farbehi et al., 2019; McLellan et al., 2020; Ruiz-Villalba et al., 2020). For example, a subpopulation of *Pdgfr*α*^*POS*^* CFs with a high expression of *Ly6a* and *Thy1* has been related to the response to interferon (Farbehi et al., 2019), whereas Muhl and colleagues describe a subpopulation of *Wif1^*POS*^/Comp^*POS*^* CFs associated with valve interstitial cells and suggest that these CFs modulate ECM in an organ and location-specific manner (Muhl et al., 2020).

This association between anatomical regions and specific fibroblast functions has been recently reported for human CFs isolated from different regions of homeostatic hearts (Litviňuková et al., 2020). In this paper, seven subpopulations of CFs are identified as based on the expression of molecules such as DCN, GSN, and PDGFRA, FB1 and FB2, showed a basal, chamber-specific fibroblast expression profile; FB3 CFs are stress-responsive and could contribute to sustain cardiac homeostasis; FB4 CFs seem to be more responsive to TGFβ signaling; and FB5 CFs are characterized by the expression of genes involved in the production, remodeling, and degradation of ECM. However, further detailed studies are needed to analyze differences in cardiac fibroblast heterogeneity between species, taking into account the technical limitations related to each one of them (e.g., in contrast to murine hearts, the isolation of the whole human CFs population is not possible, so that we have to assume that small pieces of tissue are representative enough of the complete organ).

Second, the study of CFs in the context of heart disease is of extreme relevance considering the impact of fibrosis in the progression of many cardiac ailments. Different studies have found interesting patterns of fibroblast activation in injured tissues that are associated with different functional properties and stages of disease progression in several pathologies (Frangogiannis, 2020). A perfect example of the crucial role played by CFs in disease is MI. The first 48 h after MI are characterized by a rapid inflammation and leukocyte recruitment partially promoted by CFs (Mouton et al., 2019). Following this first step, a subpopulation of fibroblast-like or stromal cells with a pro-inflammatory and pro-survival gene signature appears in the heart roughly 24 h after the damage (*IR* in Forte et al., 2020). Within the first 3 days post-infarction (dpi), several subpopulations of CFs, described by different groups, appear in the heart. In a pioneer scRNA-Seq study, a cardiac fibroblast subpopulation enriched in ECM-related genes (*Col1a1*, *Cthrc1*, *Postn*, *Fn1*, *Tnc*) was described 3 dpi (Gladka et al., 2018). These authors identified *Ckap4* gene as a marker for these activated CFs, in both mice and human cardiac ischemic samples. Between 3 and 7 dpi, several fibroblast clusters with proliferation abilities and significant expression of *Acta2*, appear near the infarct zone (Fu et al., 2018; Farbehi et al., 2019; Forte et al., 2020). In this “proliferative” period of ventricular remodeling, minor CF-like interstitial subpopulations were identified by their myeloid-phagocytic profile (Forte et al., 2020). Furthermore, a Wnt-related cluster (*Wif1*^*POS*^, *Dkk3*^*POS*^) was described and found to correspond to cells located in the scar and border zone (*WntX* in Farbehi et al., 2019). A similar cardiac fibroblast subpopulation was reported to be endocardial-derived (*EndD*, see Forte et al., 2020).

This scRNA-Seq transcriptomic study indicates that the majority of CFs responding to cardiac damage generate in the embryo from the epicardium, confirming the results from previous studies using genetic cell-tracing tools (Ruiz-Villalba et al., 2015; Moore-Morris et al., 2018). In accordance with this discovery, it is the current consensus that resident interstitial mesenchymal populations are the main source of activated CFs, and that the embryonic epicardium is the main origin of these stromal cells (Tallquist and Molkentin, 2017). The fundamental question, however, is whether all the CFs are equal. Recent studies suggest this is not the case, as shown by our own research. We have recently described a subpopulation of CFs with a crucial role in cardiac repair (*Reparative Cardiac Fibroblasts*, RCFs) that closely cluster after scRNA-Seq analysis. These RCFs appear in the infarct and border zones from 7 to 30 dpi in mice and are also present in ventricular remodeling tissues in both in pigs and human infarcted hearts. *Cthrc1* is the top marker gene of RCFs, which also express genes of the non-canonical TGFβ1/PI3K-Akt pathway (Ruiz-Villalba et al., 2020). RCFs have a gene expression pattern similar to that of other CF clusters described by Forte and colleagues, who regarded these cells as *myofibroblasts* (Forte et al., 2020). On a final note, RCFs were also detected in murine fibrotic hearts after continuous treatment with angiotensin-II and patients suffering cardiac hypertrophy (Ruiz-Villalba et al., 2020). The dynamics of these cells in both murine models of cardiac fibrosis suggests a similar role in the early stages of the pathology.

Differences in cardiac fibroblast heterogeneity are also found in the maturation phase of ventricular remodeling. A scRNA-Seq experiment has revealed a high expression of *Fstl1* in activated CFs at 14 dpi (Kretzschmar et al., 2018). At these stages of the remodeling process, the scar contained “matrifibrocytes,” a subset of activated CFs described by two different research teams (Fu et al., 2018; Forte et al., 2020). These CFs are characterized by their reduced secretory activity, restricted contractility, and low proliferative capacity, as well as by the expression of extracellular matrix and tendon related genes (such as *Comp* and *Cilp*). These data suggest that these cells are acquiring a more specialized, structural, and supporting phenotype in the mature scar (Fu et al., 2018). Other two subpopulations of activated CFs, with different transcriptomic profiles, persist in the mature scar around 30 dpi (Forte et al., 2020). These have been termed as *“late response” fibroblasts (LR)* and “matrifibrocytes” (MFC) (Figure 3).

Vascular mural (mainly smooth muscle cells, SMCs) and perivascular cells (pericytes, PCs) are abundant and important cell types in the adult heart. They are in close relation with the blood vessel endothelium and play a key role in the regulation of vascular function (Armulik et al., 2011). SMCs have a contractile phenotype traditionally characterized by the expression of *Acta2*/αSMA, *Tagln* and *Cnn1* (Yoshida and Owens, 2005; Rensen et al., 2007), while PCs are identified by their *Cspg4*/NG2 and *Pdgfrb* expression, although they have also been shown to express *Acta2* in specific locations (Nehls and Drenckhahn, 1991; O’Farrel et al., 2017). The single-cell RNA profiling of healthy heart tissues revealed that mural cells share the expression of *Cspg4*, *Pdgfrb*, *Itga7*, *Mcam*/CD146 and *Rgs5* genes (Skelly et al., 2018; Muhl et al., 2020), and that PCs, but not SMCs, express *Vtn* (Skelly et al., 2018; Farbehi et al., 2019). SMCs show less heterogeneity as compared to CFs, but they can be discretely clustered too. These important findings might be relevant to fully understand the clonal nature of atherosclerotic lesions (Bennett et al., 2016; Chappell et al., 2016), but this point has not been extensively addressed as yet (Muhl et al., 2020).

## Immune Cells

The heart comprises a small population of resident immune cells from the myeloid and lymphoid lineages (Hart and Fabre, 1981; Bönner et al., 2012; Epelman et al., 2014; Pinto et al., 2016). *Ptprc*/CD45 is a pan-leukocyte cell marker that is widely expressed in circulating/bone marrow-derived cells and has been traditionally used to also identify these cells in the heart (Nakano et al., 1990; Haudek et al., 2006; Ruiz-Villalba et al., 2015; Pinto et al., 2016). However, the characterization of bone-marrow-derived cell heterogeneity has proven to be too complex, as expected from cells deriving from multiple lineages, displaying wide functional versatility, and dynamically expressing molecular markers through time. In this section, we discuss the most relevant blood-borne cell types.

### Cardiac Monocytes/Macrophages

**FIGURE 4:**
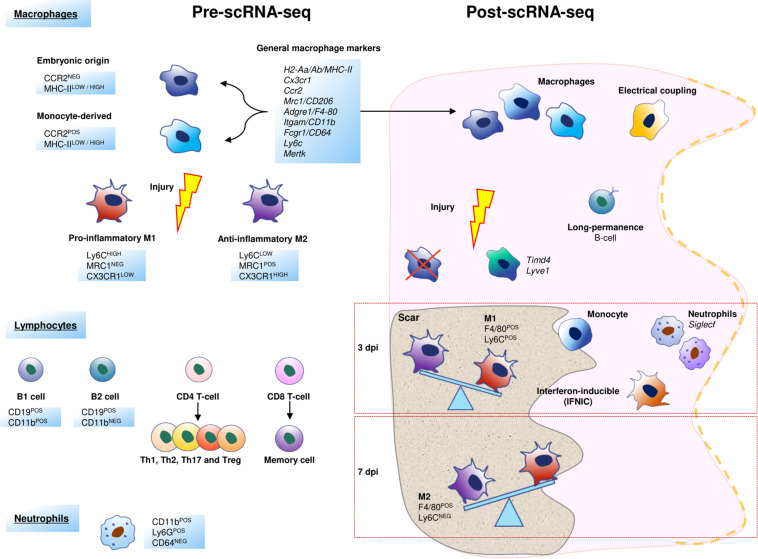
Schematic representation of the cellular heterogeneity of cardiac inflammatory cells before and after scRNA-Seq. In the scRNA-Seq part, each cell represents a cluster characterized by one or more genes, a cell function or other cell aspects. Some of these populations have been named after the original study that identified them.

Macrophages (MPs) are the epitome of cardiac immune cells. These cells can be isolated using classical markers, such as F4/80 or CD64 (Zaman et al., 2021). However, MPs can be “polarized” in response to pathologies including myocardial infarction, so that cardiac macrophage heterogeneity increases. After a cardiac injury, two classes of MPs are located in the myocardium: the classically activated M1 (Ly6C*^*HIGH*^*/MRC1*^*NEG*^*/CX3CR1*^*LOW*^*), with a pro-inflammatory profile, and the alternatively activated M2 type, which consists of anti-inflammatory and reparative MPs (Ly6C*^*LOW*^*/MRC1*^*POS*^*/CX3CR1*^*HIGH*^*) (Arnold et al., 2007; Nahrendorf et al., 2007). There is a clear trend to consider this classification as the oversimplification of a complex cellular maturation process, since unique and clear transcriptomic signatures for M1 and M2 are not evident. However, the interest in MP polarization has contributed positively to the study of the role of these cells in the adult heart (Frangogiannis, 2012; Pinto et al., 2014). During macrophage maturation, the monocyte chemoattractant protein-1 (MCP-1), and its receptor (CCR2), play an important role in the control of the process (Frangogiannis et al., 2007; Chen B. et al., 2019). Cardiac CCR2*^*NEG*^* and CCR2*^*POS*^* macrophages have distinct functional properties in both, murine and human hearts: CCR2*^*NEG*^* are considered as reparative (and are thus close to the M1 classic MP phenotype), in contrast to CCR2*^*POS*^*, which are considered as inflammatory MPs (Bajpai et al., 2018, 2019). Remarkably, a relation between cardiac macrophage heterogeneity and their embryonic origin has been pointed out: CCR2*^*NEG*^*/MHC-II*^*POS(LOW/HIGH*)^* MPs derive from embryonic progenitors during the development, and CCR2*^*POS*^*/MHC-II*^*POS(HIGH/LOW*)^* correspond to minor macrophage subpopulations derived from circulating monocytes and monocytes (Epelman et al., 2014; Lavine et al., 2014). It has been described that many resident CCR2*^*NEG*^* MPs die soon after MI (Leuschner et al., 2012; Heidt et al., 2014), but thanks to scRNA-Seq studies, it is now clear that the population of CCR2*^*NEG*^*MHC-II*^*LOW*^*TIMD4*^*POS*^*LYVE1*^*POS*^* cardiac resident MPs is maintained after injury (Dick et al., 2019; Farbehi et al., 2019; Figure 4). Interestingly, a population of LYVE1^*POS*^ tissue-resident macrophages associated with cardiovascular remodeling has been recently described in human homeostatic hearts, although they were found to be TIMD4^*NEG*^ cells (Litviňuková et al., 2020).

Remarkably, scRNA-Seq studies have yielded results that are quite similar to a macrophage cellular map resulting from the use of a set of well-known MP surface markers (*Cx3cr1*, *H2-Aa/Ab*/MHC-II, *Ccr2*, *Mrc1*/CD206, *Fcgr1*/CD64, *Adgre1*/F4/80, *Cd68*, *Itgam*/CD11b) (King et al., 2017; Skelly et al., 2018; Dick et al., 2019; Farbehi et al., 2019). However, scRNA-Seq has identified three novel subpopulations of MPs by their unique transcriptomic profile: TIMD4*^*POS*^/*LYVE-1*^*POS*^*, MHC-II*^*HIGH*^*/CCR2*^*NEG*^*, and MHC-II*^*HIGH*^*/CCR2*^*POS*^* MPs (Zaman et al., 2021). Other authors have identified only two subsets of resident MPs using scRNA-Seq in combination with cell fate mapping approaches that studied samples from several healthy organs including the heart. One of the relevant findings of this study is that one of these macrophage populations (LYVE-1*^*HIGH*^*/MHC-II*^*LOW*^*) resides in the vicinity of blood vessels, while the other one (LYVE-1*^*LOW*^*MHC-II*^*HIGH*^*) is preferentially found close to nerves (Chakarov et al., 2019).

Finally, an additional subpopulation of mononuclear cells with an intermediate M1-M2 profile has been described. This subpopulation, called interferon-inducible cells (IFNICs), is characterized by the expression of F4-80*^*POS*^*/MHC-II*^*POS*^*/CCR2*^*POS*^*/CXCL10*^*POS*^*/Ly6C*^*NEG*^*/CD11c*^*NEG*^*. IFNICs seem to have a relevant role in the amplification of inflammation 4 days after MI via the Interferon regulatory factor 3 (IRF3)/type I interferons (IFNs) axis. In an attempt to summarize multiple studies, we can conclude that only 7 macrophage subpopulations show different transcriptional profiles after the 1st week post-infarction when compared to MPs isolated from healthy hearts. In contrast, circulating MPs almost disappear from the heart around 28 days after MI, and the proportion of resident MPs is not recovered in comparison with the homeostatic heart (Dick et al., 2019). These data suggest a permanent modification in the cellular landscape because of the infarction damage. Moreover, all these findings illustrate the degree of complexity and subtlety of the maturation of MPs in the infarcted heart (King et al., 2017; Figure 4).

### Neutrophils, Dendritic, and Mast Cells: Other Myeloid Cells Found in the Heart

Single-cell transcriptomics has also revealed an increase in the heterogeneity in other inflammatory cell populations in comparison with the pre scRNA-Seq landscape (Stubbington et al., 2017; Villani et al., 2017), including cardiac resident ones. This heterogeneity is dynamic and changes in different pathological settings (Farbehi et al., 2019). After a MI, neutrophils (NPs) are the first inflammatory cells recruited in the infarcted heart, and their numbers peak between 1 and 3 dpi (Ma et al., 2013). In a very recent scRNA-Seq study, six different clusters of NPs are shown to sequentially appear after MI (Vafadarnejad et al., 2020). The two predominant NP subpopulations at 3 dpi display enrichment in *Siglecf* expression, while between 3 and 5 dpi, *Siglecf*^*HIGH*^ neutrophils represent a distinctive state, exhibiting more phagocytosis and ROS-production than other neutrophils (Figure 4).

Dendritic cells (DCs) are another relevant cell type. It is currently assumed that there are three types of DCs: plasmacytoid or pre-DCs, derived from the lymphoid lineage, and two additional types of conventional DCs (cDCs), derived from the myeloid lineage. This classification is nonetheless controversial, since DCs are known to express different sets of surface markers depending on the organ in which they reside. In the heart, the general dendritic cell population expresses *Dpp4*/CD26 and *Zbtb46* (Guilliams et al., 2016; Clemente-Casares et al., 2017), cDCs type 1 have been recently identified as CD103*^*POS*^*/CD172α*^*NEG*^*/CD11b*^*NEG*^*, and cDCs type 2 as CD103*^*NEG*^*/CD172α*^*POS*^*/CD11b*^*POS*^* (Van der Borght et al., 2017; Lee et al., 2018). Unfortunately, a plethora of bone-marrow-derived cells share some of these surface markers, limiting the unambiguous identification of these cells. In recent times, scRNA-Seq has helped to characterize CDCs in-depth (Dick et al., 2019; Farbehi et al., 2019; Martini et al., 2019). Whilst some authors propose beneficial functions of these cells regulating reparative actions from MPs and T-cells (Nagai et al., 2014; Choo et al., 2017), others have demonstrated a negative contribution of DCs to the infarct outcome (Lee et al., 2018).

Cardiac mast cells represent a minor cardiac cellular population characterized by the expression of *Fcer1g*/IgE, c-Kit/CD117, and CD9 (Sperr et al., 1994). Mast cells have been shown to increase their number and their secretion of the vasodilator and pro-fibrotic factors histamine and tryptase in failing human hearts (Patella et al., 1998). However, the role of mast cells in the mouse, the preferred animal model for the study and assessment of cells involved in the repair of the damaged heart, is not fully understood. After MI, mast cells have been reported to have a positive contribution to cardiac muscle functionality (Ngkelo et al., 2016), but have also been implicated in cardiac fibrosis (Legere et al., 2019). Thanks to a recent study using scRNA-Seq, it is now known that mast cells distributed through the epicardium and myocardium express high levels of *Mcp8* and pro-inflammatory cytokine genes like *Il6*. The level of the activation marker *Cd69* in a context of pressure overload led these authors to suggest an involvement of these cells in the early inflammatory response associated with cardiac hypertrophy (Martini et al., 2019).

### Lymphoid Cells in the Heart

Lymphocytes, comprising classic B-cells, T-cells and the innate lymphoid cells (ILCs), such as NK cells, account for a small proportion of the immune cells found in the healthy cardiac interstitium (Pinto et al., 2016). B-lymphocytes are the most frequent leukocytes in the naïve murine heart, and they are known to play a role in the modulation of inflammation and cardiac remodeling after MI (Adamo et al., 2020a). B-cells can be divided into two major populations, B-1 and B-2. Although it is still unclear which one is their origin and lineage relationship, it is known that B-2 cells (CD19*^*POS*^*CD11b*^*NEG*^*) represent the majority of cardiac lymphocytes in the adult heart (Montecino-Rodriguez and Dorshkind, 2012). Other functions have been claimed for some of these cells. For example, it was recently reported that some circulating B-cells are prone to adhere to cardiac endothelial cells and take up long-term residence in the myocardium; scRNA-Seq analysis has revealed that these lymphocytes express a distinct gene expression profile as compared with other circulating counterparts (Adamo et al., 2020a; Figure 4).

T-lymphocytes include the naïve subtypes CD4 (helper; Th) and CD8 (cytotoxic; CTL), their differentiated states such as Th1, Th2, Th17 or Treg and memory CD8^POS^ cells, and the NK cells (Zhu et al., 2010; Casey et al., 2012). These T-lymphocyte subtypes play an important role in modulating inflammation and cardiac repair (Wang et al., 2019), but disagreement exists concerning their beneficial or detrimental contribution to heart recovery after damage (Hofmann and Frantz, 2015; Li J. et al., 2020). The use of single-cell transcriptomics has revealed the existence of previously unknown states (Stubbington et al., 2017; Villani et al., 2017), but the functions of these cells are not known as yet.

## Understanding scRNA-Seq Data from a Biological Perspective

As discussed above, cardiomyocytes, endothelial, interstitial and immune cells display significant differences in gene expression. Still, it is important to note that several variables such as the genetic background, spatial location or cell-to-cell interaction may affect cell behavior and thus drive changes in the composition of cell populations (Table 1). Forte et al. (2020) have recently demonstrated that a mouse strain prone to hypertension (129S1/SvlmJ) has more susceptibility to cardiac rupture after MI than the inbred strain C57BL/6J, even if it is clear that 129S1/SvlmJ mice have more significant numbers of CFs in their ventricular walls. On the other hand, the specific cellular expression of ligands and receptors suggests the existence of molecular crosstalk patterns specific for atria or ventricles (Tucker et al., 2020; Wang et al., 2020). Moreover, the combination of single-cell and spatial transcriptomics is a powerful additional tool for the elucidation of such cellular interactions. For instance, two specific clusters of human arterial ECs and SMCs previously identified by scRNA-Seq were shown to co-localize in space and directly interact through JAG1 and NOTCH2 (Litviňuková et al., 2020). This analytical strategy based on receptor-ligand interactions can be highly relevant for the identification of paracrine signaling mechanisms existing among specific cell types, and may even suggest novel roles for CFs (Skelly et al., 2018; Litviňuková et al., 2020). The sex of individuals may also have an impact in cardiac cellular composition and susceptibility to disease (Table 1). Female mice present more CFs, T-cells and fewer granulocytes as compared with males, and these differences have gonadal hormone support (Squiers et al., 2020). Additionally, sex seems to have an impact not only on the abundance of the clusters mentioned above, but also on the gene expression profile of different cell types (McLellan et al., 2020). These differences, again, seem to be related to some degree to hormone response. Since CFs are the cell type with the highest expression of sex hormone receptors in the heart, it is very likely that these cells are the key to the articulation of such differential responses (McLellan et al., 2020). MPs have been found to differentially express genes associated with inflammation in males and genes with anti-inflammatory effects in females (Skelly et al., 2018). Of note, similar differences have been observed in human hearts. CMs and CFs were the cell types with more differentially expressed genes between the sexes (Tucker et al., 2020). In addition, ventricles from women have been found to have higher numbers of CMs than those of men (Litviňuková et al., 2020). During cardiac repair after infarct, female mice have been shown to have lower ventricular rupture rates, a higher influx of reparative leukocytes, and different regulation of immune mediators (Pullen et al., 2020). All these findings highlight the need for the evaluation of cell dynamics in both sexes, as this might have a positive impact on pharmacological interventions.

**TABLE 1:** Summary of single-cell RNA-Seq analyses performed in the adult mammalian heart.

Heart sample	Cell target	Strain	Sex	Condition	Injury stage	Platform	Cell/Nuclei sequenced number	Single-cell or nuclei	References
Complete	Cardiac cells	C57BL/6NRj, Fzt:DU	Male	Normal	–	10X Genomics	11,672	Nuclei	Galow et al., 2020
Ventricles	Cardiac cells	C57BL/6J	Not specified	Normal and ischemia reperfusion	3 dpi	SORT-seq	932	Cell	Gladka et al., 2018
Ventricles	Cardiac cells	Mixed C57BL/6J	Mixed	Neonatal, Normal and MI (LAD)	3, 7, and 14 dpi	SORT-seq	1,939	Cell	Kretzschmar et al., 2018
Atria and ventricle	Cardiac cells	Human	Female and male	Normal	–	10X Genomics	45,870/363,213	Cell and nuclei	Litviňuková et al., 2020
Complete	Cardiac cells	Human	Mixed	Normal, HF and recovery	–	ICELL8	12,266 (normal)/5,933 (HF)	Cell	Wang et al., 2020
Ventricles	Cardiac cells	C57BL/6J	Mixed	Normal and hypertension AngII-induced	14 dpi	10X Genomics	29,558	Cell and nuclei	McLellan et al., 2020
Not specified	Cardiac cells	C57BL/6J	Mixed	Normal	–	10X Genomics	>4,000	Cell	Schaum et al., 2018
Atria and ventricle	Cardiac cells	Human	Mixed	MI	–	10X Genomics	287,269	Nuclei	Tucker et al., 2020
Complete	Cardiac cells	Fzt:DU	Male	Normal	–	10X Genomics	8,635	Nuclei	Wolfien et al., 2020a
LV	CMs	C57BL/6J/Human	Male	TAC/Normal and dilated cardiomyopathy	8 weeks post-TAC/End stage	Fluidigm C1	359/116	Nuclei	See et al., 2017
Ventricles	CMs	C57BL/6J	Male	Normal and TAC	8 weeks post-infarction	ICELL8	586	Cell	Yekelchyk et al., 2019
Complete	ECs	C57BL/6J	Male	Normal	–	10X Genomics	4,612	Cell	Kalucka et al., 2020
Ventricles	ECs	Mixed C57BL/6J	Mixed	Normal and MI (LAD)	7 dpi	10X Genomics	3,200-4,000	Cell	Li et al., 2019
LV	Interstitial cells	129S4/SvJaeSor	Male	Normal and MI (LAD)	3 and 7 dpi	10X Genomics	>30,000	Cell	Farbehi et al., 2019
Ventricles	Interstitial cells	C57BL/6J and 129S1/SvlmJ	Male	Normal and MI (LAD)	1, 3, 5, 7, 14, and 28 dpi	10X Genomics	36,847	Cell	Forte et al., 2020
Ventricles	Interstitial cells	C57BL/6J	Female and male	Normal	–	10X Genomics	12,000	Cell	Skelly et al., 2018
Not specified	CFs and mural cells	C57BL/6J	Male	Normal	–	Smart-Seq2	< 6,158	Cell	Muhl et al., 2020
Ventricles	CFs	C57BL/6J	Not specified	Normal and MI (LAD)	7, 14, and 30 dpi	10X Genomics	32,669	Cell	Ruiz-Villalba et al., 2020
Complete	Immune cells	C57BL/6J	Female	Normal	–	10X Genomics	17,500	Cell	Adamo et al., 2020b
Complete	Immune cells	C57BL/6J	Not specified	Normal and MI	11 dpi	10X Genomics	1,780 (normal)/6,503 (MI)	Cell	Dick et al., 2019
Complete	Immune cells	C57BL/6J	Male	Normal and heart failure (TAC)	1- and 4-weeks post-infarction	10X Genomics	>17,853	Cell	Martini et al., 2019

*LV, left ventricle; CMs, cardiomyocyte; ECs, endothelial cell; CFs, cardiac fibroblast; MI, myocardial infarction; TAC, transverse aortic constriction; LAD, left anterior descending artery; HF, heart failure. “ – “: means no data; “dpi” means days post-infarction; “complete” indicates whole heart analysis; “cardiac cells” means the main heart cell types; “interstitial cells” do not include cardiomyocytes and “atria and ventricle” indicate separated analysis.*

## Understanding scRNA-Seq Data from a Data-Perspective

As previously shown, single-cell technologies are expanding our understanding of biological systems, and cardiac research certainly benefits from this revolution (Abplanalp et al., 2020; Ruiz-Villalba et al., 2020; Zhou and Zhang, 2020). In addition to the opportunities that this technology has made available to the scientific community, scRNA-Seq has also presented some challenges related to both experimental approaches and data-analysis routines. We discuss these aspects below.

All these technologies began with the dissociation of the tissues of interest in individual cells, a step that has been detailed in several experimental protocols in order to guarantee the reproducibility of the results. This is especially relevant in the cardiovascular field, where differences derived from the sample preparation procedure have been previously reported (Pinto et al., 2016; Zhou and Wang, 2020). After the sample preparation, cells need to be isolated, and to do this there are multiple options (Lafzi et al., 2018). Two relevant examples are the C1 microfluidic platform from Fluidigm, which isolates single cells into individual reaction chambers (Wu et al., 2014), and the Chromium platform from 10XGenomics, which uses droplets to capture single cells (Zheng et al., 2017). In both cases, cells are lysated, the RNA is reversely transcribed to complementary DNA, amplified, and processed to build up sequencing-ready libraries. There are no definitive conclusions on which protocol is the best, but criteria for selection are: “*the number of cells profiled per sample*,” “*the sequencing depth required*,” and “*tag-based – which only provides 3′ or 5′ reads – vs. full transcript sequencing*,” among others. Importantly, methods are continuously under development to improve their scale, accuracy and sensitivity, with marked differences between them (Shiroguchi et al., 2012; Brennecke et al., 2013; Grün et al., 2014; Islam et al., 2014; Ziegenhain et al., 2017). Additional considerations are the possible effects of storage (e.g., frozen samples), tissue dissociation and “single-cell vs. single-nucleus” biases (Denisenko et al., 2020). A general characteristic of the single-cell experimental protocols aimed to limit the impact of PCR-based mRNA amplification is the use of unique molecular identifies (UMI). UMIs are used to tag the fragments of mRNA (Kivioja et al., 2012; Islam et al., 2014); as a result, during the bioinformatic analysis, it is possible to characterize both mRNA and UMI signals and, therefore filter duplicate reads (identified as pairs with same mRNA sequence and same UMI).

Regarding the data-analysis part, many of the lessons learned in the analysis of RNA-Seq data (Conesa et al., 2016) need to be reviewed in the context of single-cell data (Stegle et al., 2015; Vallejos et al., 2017). Interestingly, specific aspects of bulk RNA-Seq analysis that can be imported into the single-cell RNA-Seq analysis (Soneson and Robinson, 2018). In this review we go over the different data analysis steps, with the aim of providing a broad overview on the topic and key references for the interested reader. Generally, the single-cell analysis field is advancing so rapidly that many of the state-of-the-art references and tools are published as not-peer reviewed preprints.

The very first step in the analysis of scRNA-Seq data is to generate count matrices (e.g., gene as rows and cell as columns), where every matrix cell contains the total number of mRNA reads or UMIs for a given gene and a given biological cell. The second step, *to identify what part of the data should be used for the analysis*, is (even) more crucial. It is thus necessary to *adequately* filter those genes and cells that do not provide sufficient signal or data quality. An initial filter is aimed at discarding genes with low UMIs and cells expressing a small number of genes (Ilicic et al., 2016; Soneson and Robinson, 2018). Additional filters are required, but they require us to take the nature and physiological state of the cells into consideration. For instance, cells with high levels of reads in mitochondrial genes may be dead or dying cells (Ilicic et al., 2016), but also cells at a defined metabolic state (Denisenko et al., 2020). Interestingly, it is frequent to apply “data-set or even sample-specific thresholds” to the previous filtering criteria (Luecken et al., 2020), as no standard criterion for this filtering exists (Soneson and Robinson, 2018). A third data filter is intended to identify doublets, i.e., two or more cells sharing the same cell-identifying barcode (McGinnis et al., 2019; Wolock et al., 2019; Bernstein et al., 2020; DePasquale et al., 2020). Importantly, filtering scRNA-Seq data often requires several rounds of analysis, as doublet identification requires data preprocessing and, as a result of the filtering, the data-analysis process may require to be started again.

The second step is the pre-processing of the count matrix. This step cannot be separated from the following objectives of the analysis, namely cell-subtype identification (Trapnell, 2015), differential gene expression (Soneson and Robinson, 2018), marker identification (Soneson and Robinson, 2018), or visualization (Cakir et al., 2020) among others (Stegle et al., 2015). It is not within the scope of this review to provide a comprehensive overview of all these methods, but we use the Seurat package to picture the associated steps and challenges in the analysis of scRNA-Seq data. Seurat was developed as an unified framework for scRNA-Seq analysis (Satija et al., 2015) and it has evolved to include multiple data-sets (Butler et al., 2018) and multiple data-types (Hao et al., 2020) (e.g., scATAC-Seq). In the first version, Seurat v1, the data matrix (*UMI counts per gene per cell*) was normalized in a cell-based manner as the “*number of unique UMIs per 200,000 unique UMIs*” and the data was log-transformed for the downstream analysis, which included identification of highly variable genes, Principal Component Analysis and data imputation (Satija et al., 2015). In Seurat v2 (Butler et al., 2018), the normalization was modified to “*number of unique UMIs per 10,000 unique UMIs*” and to apply natural log-transformation. Furthermore, in Seurat v2, a strategy was developed that uses Canonical Correlation Analysis (CCA) to identify the most highly correlated features of the data sets in order to align several batches (Butler et al., 2018). Seurat v3 (Stuart et al., 2019) replaced the CCA-based integrative strategy in order to include the concept of “*anchors*,” pairs of cells that can be paired across data-sets. Importantly, this anchoring strategy allows for the integration among data modalities, specifically scRNA-Seq and scATAC-Seq. Variance-stabilizing transformation to take into account the mean-variance relationship was also included in this software (Hafemeister and Satija, 2019). Seurat v4 includes a methodology for leveraging over the paired nature of multi-omic single-cell data (Hao et al., 2020). As can be concluded from this brief snapshot-based review of the Seurat package, the bioinformatic analysis pipelines are in continuous evolution, and require frequent review. Furthermore, the data pre-processing also has an impact on the differential gene expression analysis as the two steps cannot be disentangled (Soneson and Robinson, 2018).

A third challenge in the analysis of scRNA-Seq data is the identification of the different cell populations. To this end, clustering methodologies are available, as well as several systematic reviews on these procedures which might be helpful for the interpretation of the data (Duò et al., 2020; Peyvandipour et al., 2020). Once cell groups have been identified, the next step is to *label* such clusters. To do so, differential gene expression between cell groups is conducted in order to identify markers, and those markers are used to label cell groups (Zeisel et al., 2015). Manual annotation is possible, but time-consuming. Automatic annotation is the obvious alternative, but it has limitations, and its use remains an open challenge (Abdelaal et al., 2019). Fortunately, for specific tissues such as blood, the annotation strategies are maturing, and tools such as *Azimuth*^1^ (from the Satija lab) allow for the automatic annotation of peripheral blood mononuclear cells (PBMCs). Importantly, although a first draft of the cardiac cellular landscape has been described (Litviňuková et al., 2020), the use of automatic annotation in this context is still an open challenge. Another challenge is the identification of known cell subtypes within a given cell population (for instance, to identify Th1 CD4^*POS*^ T-cells within a CD4^*POS*^ T-cell population). In some cases, the data obtained may not have enough resolution (or enough number of cells) to discriminate between sub-types. A second additional challenge is the robust validation of rare cells types that can be identified because no quality control can provide sufficient evidence for these cells to be discarded (Wegmann et al., 2019).

Single-cell RNA-Seq has been frequently used for the characterization of cell differentiation processes. In this type of experiment, the samples are obtained at predetermined time-points. However, by making use of the variability of “*speeds*” of differentiation in cells, it is possible to order these cells so that such ordering can define a *pseudotime* (Trapnell et al., 2014). There are, again, many available methodologies to achieve this goal (Saelens et al., 2019) and they can be used for the characterization of complex phenomena such as hematopoiesis (Athanasiadis et al., 2017) or human heart development (Cui et al., 2019). Importantly, *pseudotime* methods are also limited in their ability to accurately order cells. Several methods have been developed as a complementary approach to estimate the dynamics of every cell by using the comparison between unspliced and spliced mRNA signal to estimate a vector of differentiation (RNA velocity) (La Manno et al., 2018). This method, known as *velocyto*, has completely changed the analysis of single-cell dynamics (e.g., SIB 2019 Bioinformatics Award), so that updated approaches for such a strategy such as *scVelo* (Bergen et al., 2020) and very recently *CellRank* (Lange et al., 2020) have appeared. At this point, we believe it is important to highlight that all these methods are powerful exploratory tools supporting model-generation approaches. Nevertheless, we cannot forget that all these tools are based on specific (mathematical) assumptions. Therefore, any new insights into biological entities or events derived from this kind of analysis will require a wet-lab validation and a careful biological interpretation (Everaert et al., 2017; Zhou and Wang, 2020).

A significant aspect of single-cell RNA-Seq data is the large number of data-points (cells) available for every sample. When every sample may contain up to 10 000 cells, and every cell contains the profile of between 2000 and 5000 genes, then “studies with many samples” enter a data-rich environment rapidly. Such an environment is ideally suited for using Machine Learning methodologies, and specific attention has been given to Deep Learning methods (Eraslan et al., 2019a). These methodologies cover a wide range of applications such as *in silico* data generation (e.g., cscGAN) (Marouf et al., 2020), data imputation (e.g., scIGANSs) (Xu et al., 2020) based on Generative Adversarial Networks (Goodfellow et al., 2014), data integration on unpaired datasets (e.g., totalVI) (Gayoso et al., 2021) or paired datasets (e.g., LIBRA) (Martinez-De-Morentin et al., 2021), among others. In summary, many of the methodologies associated with single-cell RNA-Seq analysis are using Machine Learning tools and new applications are appearing to refine many steps of the analysis framework described (Oller-Moreno et al., 2021).

Finally, while we have reviewed the most frequently used tools to analyze single-cell RNA-Seq data and discussed their limitations as well as the technical challenges that still need to be addressed in their use in a biological context, there are additional methods that may require attention (Poirion et al., 2016). Among those we would highlight: (i) data-analysis tools designed for non-bioinformaticians (Franzén and Björkegren, 2020); (ii) visualization tools for single-cell RNA-Seq (Cakir et al., 2020); (iii) identification of Gene-Regulatory Networks (Chen and Mar, 2018; van Dijk et al., 2018); (iv) new *gene expression imputation* methods, aiming at evaluating the increased sparsity observed in scRNA-Seq data (Wagner et al., 2017; Huang et al., 2018; van Dijk et al., 2018; Eraslan et al., 2019b); (v) integration of multiple (and possible massive) data-sets (Luecken et al., 2020); and (vi) the implementation of multi-omic (e.g., scRNA-Seq, Baek and Lee, 2020) and scATAC-Seq analysis (Chen H. et al., 2019; Baek and Lee, 2020). It is worth mentioning that single-cell multi-omic analysis also benefits from machine learning techniques; for instance “Latent Semantic Indexing” and “Latent Dirichlet Allocation” – both used in natural language processing – are implemented in Signac (Stuart et al., 2020) and cisTopic (Bravo González-Blas et al., 2019) scATAC-Seq packages respectively.

## Future Perspectives: Toward Data Integration

While scRNA-Seq has allowed for an unprecedented level of detail and characterization of heart cellular components, it is becoming evident that scRNA-Seq alone cannot fully disclose cardiac cell complexity (Lähnemann et al., 2020) as scRNA-Seq provides a *just-a-transcriptomical view* and additional regulatory layers are necessary to understand the system (Gomez-Cabrero et al., 2014, 2019). Fortunately, single-cell profiling is now available for chromatin accessibility (Buenrostro et al., 2015), proteomics (Cheung et al., 2020), DNA methylation (Galvão and Kelsey, 2021) or chromatin conformation assays (Ramani et al., 2020). However, the analysis of data-sets from these different analyses requires specific developments for each one of them, as well as an increased effort to provide a multi-omic integrative analysis (Jansen et al., 2019; Argelaguet et al., 2020). As the multi-omics approaches are still under development, protocols are being improved continuously, allowing for the profiling of more-than-one-omic analysis of the same cell (Chen S. et al., 2019). It is anyway clear we will have to compromise, and accept that a certain decrease in the quality of the data obtained from these approaches can be balanced with the advantages provided by the analysis of paired profiles (Lee et al., 2020).

The profiling of a cell-population is a relevant issue because it allows us to understand how cells interact and organize in space and time. Therefore, scRNA-Seq approaches can be complemented by the use of other techniques. For instance, *Spatial Transcriptomics* (ST) allows for the 2D characterization of tissue transcriptomics (Asp et al., 2019). ST and scRNA-Seq are indeed complementary, because scRNA-Seq identifies the cell-types and their markers, while ST contributes to illustrate their spatial organization within the tissue (Andersson et al., 2020). Importantly, cell-to-cell interactions can be computationally predicted by combining ligand and receptor information, their expression in each cell-type, and the available information on protein-protein interactions (Efremova et al., 2020; Hou et al., 2020). The ultimate challenge in the use of these techniques is to bring all this knowledge on cell characterization into a clinical setting (Haque et al., 2017; Keener, 2019). Therefore, it is not surprising that the cross-referencing between scRNA-Seq and other high throughput technologies, such as proteomics or metabolomics, is regarded as crucial for determining and prioritizing the molecular candidates associated with prevalent cardiac complex conditions such as heart failure (Chan et al., 2020).

## Conclusion

Despite all the relevant discoveries made using the scRNA-Seq technology, much more is needed to understand the intrinsic complexity of cell communities. We believe it is especially important to characterize in detail the temporal dimension of cell differentiation or incorporation into tissues. The paradigmatic case to illustrate this point would be that of the circulating (blood-borne) cells recruited to tissues after damage. In any case, and regardless of the biological system we choose to study via scRNA-Seq, we should always consider the conceptual limitation of the “cell marker” concept, the plasticity of molecular cell phenotypes, and all the caveats of a technology that heavily depends on bioinformatics and mathematical routines for the analysis of the data it yields. In the cardiovascular research field, scRNA-Seq has been instrumental to progress in the understanding of cardiac progenitor cell dynamics, the characterization of specific subpopulations of poorly studied cardiac cell types, and the cardiac events in which they participate. Future refinements of this knowledge are likely to derive from the improvement of protocols for cell extraction, isolation, purification, and the transcriptomic analysis itself, together with the continuous development of bioinformatic analytical tools.

## Author Contributions

EM-S and AR-V contributed to the conception, designed the study, and wrote the first draft of the manuscript. XM, JP-P, and DG-C wrote sections of the manuscript. All authors contributed to manuscript revision, read, and approved the submitted version.

## Conflict of Interest

The authors declare that the research was conducted in the absence of any commercial or financial relationships that could be construed as a potential conflict of interest.

## Footnotes

1 https://satijalab.org/azimuth/
